# Effects of Different Diluents and Freezing Methods on Cryopreservation of Hu Ram Semen

**DOI:** 10.3390/vetsci11060251

**Published:** 2024-06-03

**Authors:** Liuming Zhang, Xuyang Wang, Caiyu Jiang, Tariq Sohail, Yuxuan Sun, Xiaomei Sun, Jian Wang, Yongjun Li

**Affiliations:** Key Laboratory for Animal Genetics and Molecular Breeding of Jiangsu Province, College of Animal Science and Technology, Yangzhou University, Yangzhou 225009, China; 18352767281@163.com (L.Z.); 18705271593@163.com (X.W.); m19352674787@163.com (C.J.); drtariqsohail34@yahoo.com (T.S.); 19802622001@163.com (Y.S.); xiaomeisun_yz@163.com (X.S.); jianwang1223@163.com (J.W.)

**Keywords:** diluent, freezing methods, sperm motility, sperm functional integrity, ROS level

## Abstract

**Simple Summary:**

Ram sperm are easily affected by cold shock and ice crystal damage during cryopreservation. Appropriate dilutions and freezing methods are crucial as they can provide energy to sperm, maintain a stable environment, minimize the formation of ice crystals, and ultimately impact the cryopreservation of semen. However, there is a lack of diluent formulas and semen freezing methods with simple components, low cost, easy operation, and effective results. Therefore, the effects of eight basic diluents and three freezing methods during ram semen cryopreservation were studied by assessing sperm motility, biokinetic characteristics, the reactive oxygen species (ROS) level, and acrosome and membrane integrity. The findings indicated that combining basic diluent C (Tris-Citric acid-fructose) with liquid nitrogen fumigation (2 cm above the liquid–gas interface for 20 min) achieved the best effect for semen cryopreservation.

**Abstract:**

The purpose of this study was to investigate the effects of different diluents and freezing methods on the quality of thawed sperm after cryopreservation and find an inexpensive and practical method for freezing Hu ram semen for use in inseminations under farm conditions. Ejaculates were collected from five Hu rams. In experiment I, ejaculates were diluted with eight different freezing diluents (basic diluents A, B, C, D, E, F, G, and H). After dilution and cooling, the samples were loaded into 0.25 mL straws and frozen using the liquid nitrogen fumigation method. In experiment II, diluent C was used as the basic diluent and the semen was frozen using liquid nitrogen fumigation and two program-controlled cooling methods. For analysis, frozen samples were evaluated in terms of motility parameters (total motility (TM), progressive motility (PM)), biokinetic characteristics (straight-line velocity (VSL), average path velocity (VAP), curvilinear velocity (VCL), amplitude of lateral head displacement (ALH), wobble movement coefficient (WOB), average motion degree (MAD)), reactive oxygen species (ROS) level, and membrane and acrosome integrity. In experiment I, diluent C had higher TM, PM, and acrosome and membrane integrity and lower ROS compared to other extenders (*p* < 0.05) except diluent A. Diluent C exhibited higher (*p* < 0.05) VCL, VAP, ALH, WOB, and MAD compared to diluents B, D, E, and F. In experiment II, TM and all biokinetic characteristics did not show significant differences (*p* > 0.05) amongst the three freezing methods. Liquid nitrogen fumigation resulted in higher (*p* < 0.05) PM, membrane integrity, acrosome integrity, and lower ROS level compared to the program. In conclusion, the thawed semen diluted with diluent C had higher quality compared to other diluents. The liquid nitrogen fumigation demonstrated superior semen cryopreservation effects compared to the program-controlled cooling method using diluent C.

## 1. Introduction

In China, high-quality breeding stock has gradually played an important role in the intensive development of modern animal husbandry. The Hu sheep is a first-class protected breed in China and a rare white lambskin sheep breed in the world [1]. The Hu sheep are highly valued by farmers for their high fertility and continuous estrus cycles and have the advantages of high temperature and high humidity resistance, strong adaptability, and multiple lambs per litter [2]. Hu sheep have a high slaughter rate and excellent meat quality, being rich in protein [3]. At the same time, they have a very broad prospect in the processing of lamb products and meat products [4]. Therefore, the scale of Hu sheep breeding is expanding rapidly. Artificial insemination (AI) and semen cryopreservation technologies play a crucial role in large-scale farming. Semen cryopreservation is a commonly used reproductive technology in animal husbandry and an important part of AI technology [5].

Semen cryopreservation involves storing collected semen in liquid nitrogen after dilution, cooling, and equilibration to inhibit sperm metabolism and achieve long-term preservation [6]. After thawing and warming, sperm can regain partial motility and maintain normal physiological functions and fertilization capacity [7]. Semen cryopreservation enables AI to overcome limitations of time and distance, utilize semen from high-quality breeding bulls, lower breeding expenses, and enhance reproductive efficiency [8]. At the same time, semen cryopreservation can also reduce the spread of disease and has important implications for the protection of endangered species [9].

For semen cryopreservation technology, the process involved in semen diluents and freezing methods is very important, as it can directly affect the cryopreservation effectiveness of semen [10,11]. During the cooling, freezing, and thawing process, diluent provides energy for sperm, reduces freezing damage, and increases sperm volume [12,13]. Semen freezing methods mainly include liquid nitrogen fumigation and program-controlled cooling [14]. The liquid nitrogen fumigation method requires less instrumentation and is especially economical and convenient when the number of frozen tubes is small [15]. The program-controlled cooling method can control the cooling rate by controlling the amount of liquid nitrogen vapor injection, which has the advantages of temperature control and stable quality of frozen sperm [16]. However, it has not been widely used in production due to its dependence on programmable temperature controllers, complicated and time-consuming operation, and high investment cost. Therefore, liquid nitrogen fumigation may still be the main method of freezing semen due to its advantages of simplicity and low cost. Therefore, a simple and effective diluent and freezing method are important factors that influence the freezing effect of semen. However, there are few studies on frozen diluents and freezing methods for semen at present and the freezing effect is also different. The quality of thawed semen may not meet the production requirements. Falchi [17] used a basic diluent of Tris-Citric acid-glucose and liquid nitrogen fumigation in the freezing study of buck semen and the thawed sperm total motility (TM, %) was 61%. Igbokwe [18] used a Tris-based diluent and liquid nitrogen fumigation in the freezing study of goat semen and the thawed sperm motility was 36%. İnanç [19] used a basic diluent of Tris-Citric acid-fructose and liquid nitrogen fumigation in the freezing study of Sonmez ram semen and the sperm TM after thawing was 28%. Pradiee [20] used a basic diluent of Tris-Citric-Glucose acid and liquid nitrogen fumigation in the freezing study of mouflon ram semen and the thawed sperm TM was 15%. Vozaf [21] used a commercial diluent (Triladyl^®^) and program-controlled cooling in the freezing study of Wallachian ram semen and the thawed sperm TM was 62%. Therefore, the aim of this study was to evaluate the effects of different diluents and freezing methods on the quality parameters of thawed Hu ram semen.

## 2. Materials and Methods

### 2.1. Experimental Design

The primary objective of experiment I was to investigate the impact of different basic diluents (A, B, C, D, E, F, G, and H) on the thawed sperm motility parameters, biokinetic characteristics, reactive oxygen species (ROS), and membrane and acrosome integrity. The experiment II was designed to investigate the effect of different freezing methods (program 1, program 2, and liquid nitrogen fumigation) on the thawed sperm motility parameters, biokinetic characteristics, ROS, and membrane and acrosome integrity based on the diluent C.

### 2.2. Animals and Semen Collection

All the animals were in good health and accommodated at the sheep facility located at Yangzhou University under the same management conditions. Specifically, 2–3-year-old rams were fed a specific amount of concentrate and alfalfa grass daily and drank freely. An artificial vagina was used to collect semen three times a week. Overall, 80 ejaculations were collected from five Hu rams whose fertility had already been checked between June and August. The samples were examined for semen quality. Only those with a volume between 0.5–1.5 mL, a concentration greater than 2.5 × 10^9^ sperm/mL, and TM greater than 80% per ejaculation were considered normal and pooled evenly for subsequent experiments.

### 2.3. Preparation of Semen Diluents

Six different basic diluents, A, B, C, D, E, and F were prepared in the laboratory as shown in Table 1. Among them, the diluent E was the phosphate-buffered solution (PBS) and F was normal saline (NS). In addition, the diluents G (Shengyuan, Zhengzhou, China) and H (Beiteshuang, Beijing, China) were commercial diluents without antifreeze protectants. The freezing extender I was composed of 20% egg yolk and 80% basic diluent, while extender II was composed of 6% and glycerol 94% freezing extender I. The basic diluent can be stored at 4 °C and the egg yolk and glycerol should be added before use.

### 2.4. Cryopreservation and Thawing of Sperm

The semen sample and freezing extender I were diluted in a 1:3 ratio. Semen were enveloped in a towel and subjected to cooling at 4 °C for a duration of 2.5 h. Subsequently, freezing extender II was introduced at a proportion of 1:2, following which the semen was allowed to stabilize at a temperature of 4 °C for an additional duration of 2.5 h. Subsequently, all semen samples were loaded into 0.25 mL straws and sealed with sealing powder.

For the liquid nitrogen fumigation, the straws were placed in the vapors of liquid nitrogen for 20 min, positioned at a distance of 2 cm above the surface of the liquid, and then immediately immersed in the liquid nitrogen for storage. For the program-controlled cooling, the temperature is decreased at a controlled rate in three steps. The straws were placed into a programmable temperature controller (TF-PA-II, Tianfeng Industrial, Shanghai, China). For the program 1, the temperature was initially reduced at 5 °C/min from 4 °C to −10 °C, then at 40 °C/min from −10 °C to −100 °C, and finally at 20 °C/min from −100 °C to −140 °C. For the program 2, the temperature was initially reduced at 5 °C/min from 4 °C to −10 °C, then at 20 °C/min from −10 °C to −100 °C, and finally at 20 °C/min from −100 °C to −140 °C. After the programs cooled down, the straws were immediately immersed in liquid nitrogen for storage.

After one week, the frozen straws were randomly selected and thawed for 8 s at 55 °C in a water bath before being prepared for subsequent analyses.

### 2.5. Evaluation of Sperm Motility and Biokinetic Characteristics

The thawed sperm motility and biokinetic characteristics were evaluated by a CASA system (ML-608JZ II Mailang, Nanning, China). Briefly, the thawed semen was diluted in basic diluent (1:4.5) and a volume of 1.4 µL was applied to a chamber designed for counting sperm (YA-1, Yucheng, Nanjing, China). Various parameters such as TM, progressive motility (PM, %), straight-line velocity (VSL, μm/s), average path velocity (VAP, μm/s), curvilinear velocity (VCL, μm/s), amplitude of lateral head displacement (ALH, μm), wobble movement coefficient (WOB, %), and average motion degree (MAD, °/s) were examined by studying at least 500 sperm in various microscopic fields using the CASA.

### 2.6. Evaluation of Sperm Membrane Integrity

**Figure 1:**
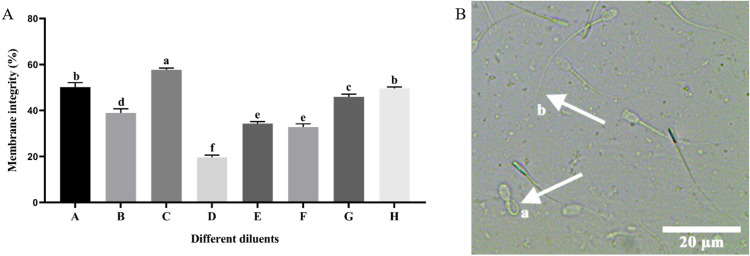
Evaluation of the membrane integrity. (**A**) Effect of different diluents on the thawed sperm membrane integrity. Note: A different letter indicates significant differences (*p* < 0.05) and the identical letter denotes the absence of significant distinctions (*p* > 0.05). (**B**) Microscopic result of sperm in the evaluation of membrane integrity. Sperm that are curled indicate an intact membrane (a arrow), while sperm that are straight indicate a damaged membrane (b arrow).

The thawed sperm membrane integrity was evaluated by a hypo-osmotic swelling test (HOST) [22]. A 20 µL aliquot of thawed semen was diluted in 200 µL of hypo-osmotic solution (9 mg/mL fructose and 4.9 mg/mL sodium citrate). The mixture was incubated at a temperature of 37 °C for 30 min. Subsequently, a phase contrast microscope with magnification (CX31, Olympus, Tokyo, Japan) of 400× was used for the evaluation and 200 sperm (curled and straight) were counted. As shown in Figure 1B, curled sperm (a) had an intact membrane, while the membranes of the straight ones (b) were impaired. 

### 2.7. Evaluation of Sperm Acrosome Integrity

The thawed sperm acrosome integrity was evaluated by fluorescence staining with PI (Solarbio, Beijing, China) and FITC-PNA (Sigma, Saint Louis, MO, USA) [23]. Briefly, the thawed semen was diluted in basic diluent (1:6) and 100 µL of samples were combined with FITC-PNA at a concentration of 200 µg/mL, along with 2 µL of PI at a concentration of 0.5 mg/mL. The mixture was placed in an incubator set at a temperature of 37 °C for 10 min in the dark. After the addition of 700 μL of PBS, the assay was performed with a FACS caliber flow cytometer (Beckman Coulter, Shanghai, China). The sperm with FITC^−^/PI^−^ and FITC^−^/PI^+^ were classified as having an intact acrosome and 10,000 sperm were detected by flow cytometry. 

### 2.8. Evaluation of Sperm ROS Level

The level of ROS production in the sperm was evaluated by a fluorescent probe with 2,7-dichlorodi-hydrofluorescein diacetate (DCFH-DA, Beyotime, Shanghai, China) [24]. Briefly, the thawed semen was diluted in basic diluent (1:6) and 50 µL of samples were mixed with DCFH-DA (10 mM). The combination was placed in an incubator set at a temperature of 37 °C for a duration of 30 min while being shielded from light exposure. After the end of the incubation, wash with PBS and resuspend with the addition of 400 μL of PBS. A multi-mode microplate reader (PerkinElmer, Waltham, MA, USA) was used to detect the fluorescence intensity at 488 nm excitation and 525 nm emission and the ROS level was expressed with fluorescence intensity.

### 2.9. Statistical Analysis

The data were analyzed using the SPSS (version 25.0, IBM, Armonk, NY, USA, 2017) software. One-way ANOVA was used for data analysis and the difference among means was tested by Duncan’s multiple range test. *p* < 0.05 was considered as significant and the results for all parameters were expressed as Mean ± SEM. Each group was duplicated four times.

## 3. Results

### 3.1. Effect of Different Diluents on Sperm Motility and Biokinetic Characteristics during Cryopreservation

As indicated in Table 2, the TM and MAD of thawed sperm in diluents A and C were greater (*p* < 0.05) compared to the other diluents. The thawed PM of sperm preserved in diluent C exhibited a statistically significant increase (*p* < 0.05) compared to the other diluents. The thawed PM of sperm preserved in diluents A and H was significantly higher (*p* < 0.05) than that of diluents B, D, E, F, and G. The thawed VCL, VAP, and ALH of sperm preserved in diluents A, C, G, and H exhibited a statistically significant increase (*p* < 0.05) compared to the other diluents. The thawed WOB of sperm preserved in diluents A and C exhibited a statistically significant increase (*p* < 0.05) compared to the diluents B, D, E, F, and G.

### 3.2. Effect of Different Diluents on Sperm Membrane Integrity during Cryopreservation

As indicated in Figure 1, the thawed membrane integrity of sperm preserved in diluent C exhibited a statistically significant increase (*p* < 0.05) compared to the other diluents. The thawed membrane integrity of sperm preserved in diluents A and H exhibited a statistically significant increase (*p* < 0.05) compared to diluents B, D, E, F, and G. However, there was no statistically significant difference observed between the diluents A and H (*p* > 0.05). The thawed sperm membrane integrity of diluent B exhibited a statistically significant increase (*p* < 0.05) compared to the diluents D, E, and F. The thawed sperm membrane integrity of diluent D exhibited a statistically significant decrease (*p* < 0.05) compared to the other diluents.

### 3.3. Effect of Different Diluents on Sperm Acrosome Integrity during Cryopreservation

**Figure 2:**
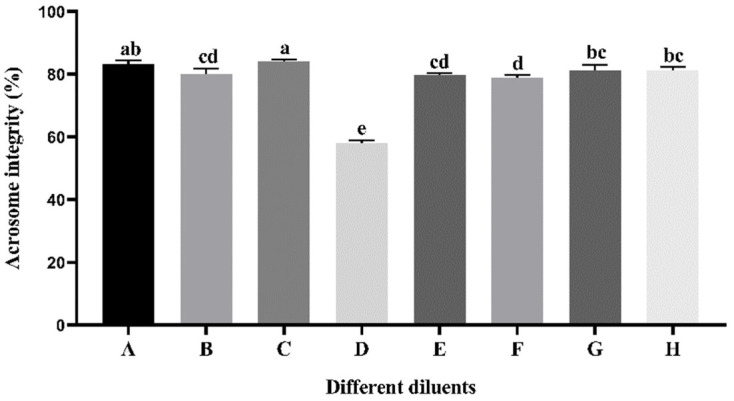
Effect of different diluents on the thawed acrosome integrity of sperm. Note: A different letter indicates significant differences (*p* < 0.05) and the identical letter denotes the absence of significant distinctions (*p* > 0.05).

As indicated in Figure 2, the thawed acrosome integrity of sperm preserved in diluent C was significantly higher (*p* < 0.05) than that in the other diluents except for diluent A (*p* > 0.05). The thawed sperm acrosome integrity of diluent A was significantly higher (*p* < 0.05) than that of diluents B, D, E, and F. The thawed acrosome integrity of sperm preserved in diluents G and H exhibited a statistically significant increase (*p* < 0.05) compared to the diluents D and F. The thawed sperm acrosome integrity of diluent D exhibited a statistically significant decrease (*p* < 0.05) compared to the other diluents.

### 3.4. Effect of Different Diluents on Sperm ROS Level during Cryopreservation

**Figure 3:**
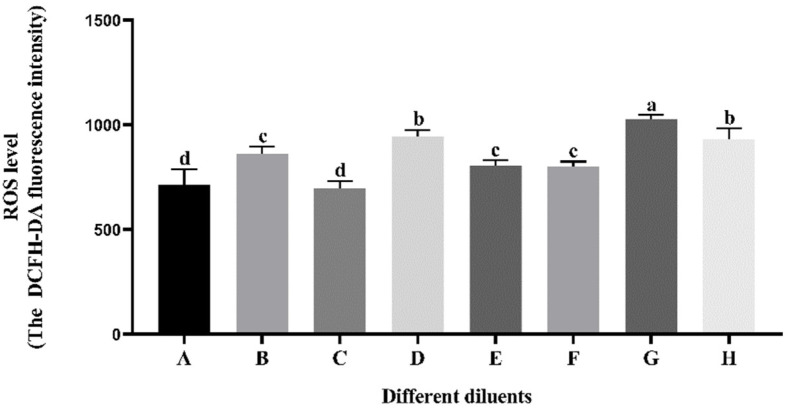
Effect of different diluents on the thawed sperm ROS level. Note: A different letter indicates significant differences (*p* < 0.05) and the identical letter denotes the absence of significant distinctions (*p* > 0.05). ROS: reactive oxygen species, DCFH-DA: 2,7-dichlorodi-hydrofluorescein diacetate.

As indicated in Figure 3, the thawed ROS level of sperm preserved in diluents A and C was significantly lower (*p* < 0.05) than that of the other diluents but there was no significant difference between the two groups (*p* > 0.05). The thawed ROS level of sperm preserved in diluents B, E, and F exhibited a statistically significant decrease (*p* < 0.05) compared to the diluents D, G, and H. However, there was no significant difference (*p* > 0.05) between diluents B, E, and F.

### 3.5. Effect of Different Freezing Methods on Sperm Motility and Biokinetic Characteristics during Cryopreservation

As indicated in Table 3, the thawed TM of sperm from the liquid nitrogen fumigation group was the highest (*p* > 0.05) compared to programs 1 and 2. The thawed PM of sperm from the program 1 and liquid nitrogen fumigation groups was significantly higher (*p* < 0.05) than that of the program. There was no significant difference (*p* > 0.05) among the three groups in sperm biokinetic characteristics.

### 3.6. Effect of Different Freezing Methods on Sperm Membrane and Acrosome Integrity during Cryopreservation

**Figure 4:**
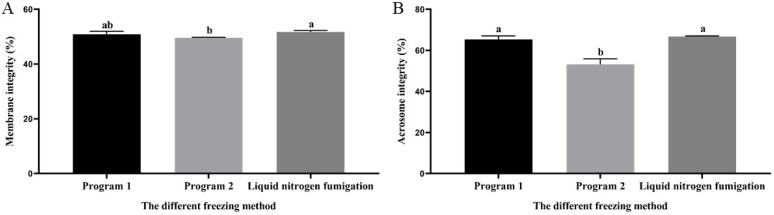
Effect of different freezing methods on the thawed sperm membrane and acrosome integrity. (**A**) Sperm membrane integrity and (**B**) sperm acrosome integrity. Note: A different letter indicates significant differences (*p* < 0.05) and the identical letter denotes the absence of significant distinctions (*p* > 0.05).

As indicated in Figure 4A, the thawed membrane integrity of sperm from the liquid nitrogen fumigation group was significantly higher (*p* < 0.05) than that of program 2 but there was no significant difference (*p* > 0.05) from the program. As indicated in Figure 4B, the thawed acrosome integrity of sperm from the program 1 and liquid nitrogen fumigation groups exhibited a statistically significant decrease (*p* < 0.05) compared to the program 1 but there was no statistically significant variance observed between the two cohorts (*p* > 0.05).

### 3.7. Effect of Different Freezing Methods on the Sperm ROS Level during Cryopreservation

**Figure 5:**
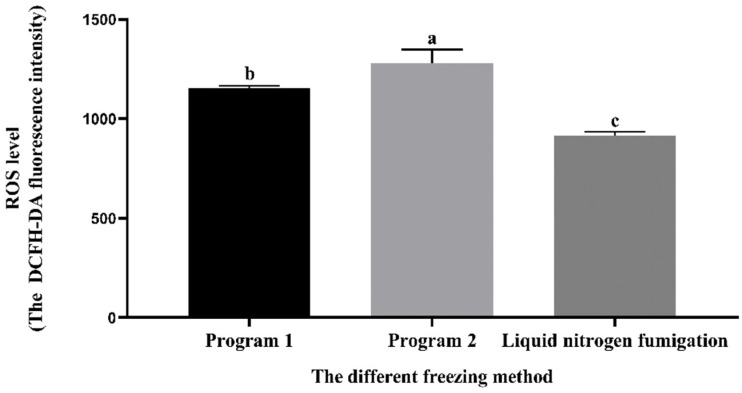
Effect of different freezing methods on the thawed sperm ROS level. Note: A different letter indicates significant differences (*p* < 0.05) and the identical letter denotes the absence of significant distinctions (*p* > 0.05).

As indicated in Figure 5, the thawed ROS level of sperm from the liquid nitrogen fumigation group exhibited a statistically significant decrease (*p* < 0.05) compared to the other programs. The thawed ROS level of sperm from the program 1 group was significantly lower (*p* < 0.05) than that of program 2.

## 4. Discussion

The development of a simple and effective semen diluent and cryopreservation technique is essential for the implementation of AI, preserving biodiversity and reducing inbreeding. Various factors influence the effect of semen cryopreservation including diluent composition, dilution ratios, equilibration time, freezing method, thawing temperature, and time [25]. In this study, the effects of various basic diluents (A, B, C, D, E, F, G, and H) and three freezing methods on the thawed sperm quality of Hu ram semen were investigated. The results of this research provide clear evidence that the liquid nitrogen fumigation method (at a height of 2 cm above liquid nitrogen for 20 min) and basic diluent C (Tris-Citric acid-fructose based) supplemented with 20% egg yolk and 6% glycerol are effective, cost-efficient, superior, and simple cryopreservation methods for the challenging sperm of rams compared to other diluents and the program-controlled cooling method.

In the process of semen freezing, the appropriate diluent composition can provide energy for sperm, provide a suitable living environment, and minimize the damage caused by freezing to sperm [26]. The components of semen diluents generally include buffering substances such as Tris, citric acid, and sodium citrate, antifreeze protectors like egg yolk and glycerol, and sugary substances such as fructose and glucose [27]. The TM, PM, biokinetic characteristics, membrane, and acrosome integrity of cryopreserved sperm in diluents E and F exhibited a statistically significant decrease (*p* < 0.05) compared to diluents A and C. This showed that it was not enough to only contain egg yolk and glycerol in the diluent. In this study, we found that buffers such as Tris and citric acid along with carbohydrates were crucial in semen freezing, which could directly affect the cryopreservation of semen. Bravo’s [28] research showed that the semen cryopreservation effect of Tris-based diluent was much higher than that of PBS group. Tris acted as a good buffer in the diluent. Graham’s [29] research on bovine sperm concluded that Tris was the most ideal buffer in semen freezing. Metabolites produced during sperm metabolism can alter the pH of the environment, leading to the inhibition of certain enzyme activities [30]. Substances like citric acid and sodium citrate could help maintain pH stability in the environment. Carbohydrates are substances that not only provide energy for sperm but also prevent structural and substructural damage to sperm during cryopreservation [31,32]. Compared to diluent C, diluent B had a better preservation effect at low temperature [33]. However, the thawed semen diluted with diluent B had lower (*p* < 0.05) TM, PM, biokinetic characteristics, and membrane and acrosome integrity and higher (*p* < 0.05) ROS levels compared to diluent C. The difference might be attributed to the cryostimulation causing more damage to sperm and the protective nature of the two buffers Tris and citric acid in diluent C compared to the single buffer sodium citrate in diluent B [34]. Diluent D was found to be less effective in cryopreservation compared to the other diluents. This might be because the content of the buffer substance was too small to maintain the stability of the diluent. At the same time, Lv’s [35] research demonstrated that adding citric acid to the diluent had a more effective semen-freezing effect compared to using sodium citrate. Diluent C had higher (*p* < 0.05) PM and membrane integrity in comparison to diluent A, while demonstrating no statistically significant variance (*p* > 0.05) in other parameters. This might be because the utilization of fructose by Hu sheep was more efficient than that of glucose. Previous studies showed that fructose was better than glucose in semen preservation in buck [36] and wolf [37]. Diluent C exhibited higher (*p* < 0.05) TM, PM, MAD, membrane integrity, and acrosome integrity, as well as lower (*p* < 0.05) ROS levels compared to two commercial diluents. The specific components of the two commercial diluents were not clear. Probably because there was not a universal diluent, they are suitable for both sheep and goats and not specific to particular species and breeds. As a result, the impact of semen cryopreservation might diminish. Therefore, the composition of the frozen diluent played a crucial role in preserving the motility of sperm during freezing and thawing. Avdatek [38] and Bucak [39] used the same basic diluent as ours in the study of semen freezing of crossbred sheep and Merino ram but the freezing method was different and the thawed sperm PM was only 13% and 10%. These results showed that only suitable dilution combined with a correct freezing method can achieve a good freezing effect.

During the process of semen dilution and cryopreservation, sperm will be damaged to varying degrees by the change in external environmental temperature. During the freezing process, sperm are most vulnerable to damage within the temperature range of 0 to −60 °C, also known as the dangerous temperature zone [40]. In this temperature range, affected by the cooling rate, ice crystals might form, causing irreversible damage to sperm [41]. Program 2 showed lower (*p* < 0.05) PM compared to program 1 and the liquid nitrogen fumigation method. This might be due to the slow cooling rate of program 2 compared to the liquid nitrogen fumigation, resulting in the production of a large number of harmful ice crystals, which damaged the sperm and caused a decrease in sperm PM. In the process of semen freezing, damage to any part of the membrane will affect the function of sperm and result in the loss of fertilization ability [42]. The sperm acrosome contains numerous hydrolytic enzymes that are essential for sperm to penetrate cumulus cells and the zona pellucida during acrosome reactions [43]. At the same time, the formation of ice crystals can also lead to the denaturation of lipoproteins, damaging the structure of the sperm membrane, which in turn leads to the loss of material in the parietal body [44]. The liquid nitrogen fumigation method showed higher (*p* < 0.05) membrane and acrosome integrity compared to program 2 and it did not have a significant difference (*p* > 0.05) compared to the program. Under normal conditions, sperm could maintain the balance of ROS; physiological concentrations of ROS play a crucial role in sperm capacitation and acrosome response but an excessive amount of ROS could lead to damage [45]. Program 2 had a higher (*p* < 0.05) ROS level compared to program 1 and liquid nitrogen fumigation. This might be due to the slow cooling rate of program 2, which led to ice crystal damage and reduced sperm motility and caused the release of ROS from dead sperm [46]. Stuart’s [47] research showed that the quality of semen obtained by rapid freezing was higher than that by slow freezing, which was also consistent with the results of this study. A study by Galarza [48] on cryopreservation of Merino ram semen reported thawed TM 61% using the program-controlled cooling method. In the study of Iberian ibex semen, Esteso [49] found that compared with liquid nitrogen fumigation, the quality of frozen semen obtained by a program-controlled cooling method is higher. These results showed that the quality of frozen semen was closely related to the variety, diluent composition, and freezing method. On the other hand, liquid nitrogen fumigation was cheaper, less costly, and easier to carry out than programmed cooling and was therefore more widely used worldwide. In conclusion, combining diluent C with liquid nitrogen fumigation could be applied in field conditions without the need for a complex dilution formula or costly equipment, which could significantly increase the utilization rate of high-quality breeding bulls, accelerate the process of breed improvement, and enhance breeding efficiency.

## 5. Conclusions

Based on the results of our study regarding CASA, membrane integrity, and acrosome integrity and ROS level, we concluded that thawed semen from Hu ram previously diluted with diluent C exhibited higher quality compared to other diluents. On the other hand, the findings revealed that freezing methods based on the diluent C showed that liquid nitrogen fumigation could achieve better results in semen cryopreservation compared to the program-controlled cooling method. Moreover, compared to the program-controlled cooling method, the liquid nitrogen fumigation method is simpler to operate and can achieve superior results without requiring expensive equipment. However, in vitro fertilization trials or AI may be required in the future to further verify the effectiveness of the diluent and freezing methods.

## Figures and Tables

**Table 1:** Six different diluent formulations.

Constituent	A	B	C	D	E(PBS)	F(NS)
Tris (Sangon Biotech, Shanghai, China)	1.82 g	-	1.82 g	0.28 g	-	-
Glucose (Sangon Biotech, Shanghai, China)	0.25 g	-	-	0.25 g	-	-
Fructose (Sangon Biotech, Shanghai, China)	-	0.25 g	0.25 g	-	-	-
Citric acid (Sangon Biotech, Shanghai, China)	0.91 g	-	0.91 g	-	-	-
Sodium citrate (Sangon Biotech, Shanghai, China)	-	1.20 g	-	0.35 g	-	-
Sodium bicarbonate (Sangon Biotech, Shanghai, China)	-	-	-	0.05 g	-	-
Sodium chloride (Sangon Biotech, Shanghai, China)	-	-	-	-	400.33 mg	0.45 g
Potassium chloride (Sangon Biotech, Shanghai, China)	-	-	-	-	10.06 mg	-
Dibasic Sodium Phosphate (Sangon Biotech, Shanghai, China)	-	-	-	-	56.78 mg	-
Potassium dihydrogen phosphate (Sangon Biotech, Shanghai, China)	-	-	-	-	12.25 mg	-
Pen Strep (Thermo, Waltham, MA, USA)	10,000 IU	10,000 IU	10,000 IU	10,000 IU	10,000 IU	10,000 IU
Total volume	50 mL	50 mL	50 mL	50 mL	50 mL	50 mL

**Table 2:** Effect of different diluents on sperm motility and biokinetic characteristics.

Different Diluents	TM (%)	PM (%)	VSL (µm/s)	VCL (µm/s)	VAP (µm/s)	ALH (µm)	WOB (%)	MAD (°/s)
A	74.82 ± 1.22 ^a^	60.32 ± 1.02 ^b^	40.98 ± 0.93 ^b^	60.33 ± 1.73 ^a^	42.66 ± 1.22 ^a^	17.67 ± 0.51 ^a^	0.53 ± 0.02 ^a^	39.10 ± 2.60 ^a^
B	54.09 ± 0.44 ^d^	39.56 ± 1.01 ^d^	41.12 ± 1.24 ^b^	53.77 ± 0.62 ^b^	38.02 ± 0.44 ^b^	15.75 ± 0.18 ^b^	0.39 ± 0.04 ^bc^	20.75 ± 0.82 ^c^
C	76.81 ± 1.04 ^a^	64.42 ± 0.84 ^a^	41.18 ± 1.41 ^b^	62.05 ± 0.52 ^a^	43.88 ± 0.37 ^a^	18.18 ± 0.15 ^a^	0.58 ± 0.06 ^a^	43.54 ± 4.79 ^a^
D	3.81 ± 0.14 ^g^	2.57 ± 0.21 ^g^	29.14 ± 2.46 ^c^	43.71 ± 1.14 ^c^	30.90 ± 0.81 ^c^	12.80 ± 0.34 ^c^	0.36 ± 0.02 ^c^	2.78 ± 0.27 ^e^
E	39.12 ± 0.49 ^e^	24.98 ± 0.92 ^e^	40.54 ± 1.90 ^b^	53.20 ± 0.99 ^b^	37.62 ± 0.70 ^b^	15.58 ± 0.29 ^b^	0.36 ± 0.01 ^c^	11.44 ± 0.27 ^d^
F	31.47 ± 1.79 ^f^	19.38 ± 0.90 ^f^	40.88 ± 1.00 ^b^	53.44 ± 0.95 ^b^	37.79 ± 0.68 ^b^	15.66 ± 0.28 ^b^	0.40 ± 0.04 ^bc^	9.60 ± 0.14 ^de^
G	66.37 ± 2.78 ^c^	51.57 ± 0.88 ^c^	47.65 ± 0.30 ^a^	60.43 ± 0.51 ^a^	42.73 ± 0.36 ^a^	17.70 ± 0.15 ^a^	0.36 ± 0.02 ^c^	28.21 ± 2.44 ^b^
H	70.68 ± 0.82 ^b^	57.92 ± 1.37 ^b^	43.88 ± 0.19 ^b^	60.39 ± 0.75 ^a^	42.70 ± 0.53 ^a^	17.69 ± 0.22 ^a^	0.48 ± 0.03 ^ab^	29.61 ± 2.21 ^b^

Note: A different letter in each column indicates significant differences (*p* < 0.05) and the identical letter denotes the absence of significant distinctions (*p* > 0.05). TM: total motility, PM: progressive motility, VSL: straight-line velocity, VCL: curvilinear velocity, VAP: average path velocity, ALH: amplitude of lateral head displacement, WOB: wobble movement coefficient, MAD: average motion degree.

**Table 3:** Effect of different freezing methods on sperm motility and biokinetic characteristics.

Different Freezing Method	TM (%)	PM (%)	VSL (µm/s)	VCL (µm/s)	VAP (µm/s)	ALH (µm)	WOB (%)	MAD (°/s)
Program 1	66.72 ± 1.36	54.62 ± 0.98 ^a^	40.00 ± 0.46	60.10 ± 0.81	42.50 ± 0.57	17.60 ± 0.24	0.53 ± 0.01	32.51 ± 3.57
Program 2	64.09 ± 2.42	50.58 ± 0.89 ^b^	39.60 ± 0.51	58.81 ± 0.64	41.58 ± 0.45	17.23 ± 0.19	0.52 ± 0.03	36.89 ± 5.85
Liquid nitrogen fumigation	69.24 ± 0.58	55.52 ± 0.55 ^a^	40.27 ± 0.76	60.56 ± 1.24	42.82 ± 0.87	17.74 ± 0.36	0.56 ± 0.01	36.77 ± 1.99

Note: A different letter in each column indicates significant differences (*p* < 0.05) and the identical letter denotes the absence of significant distinctions (*p* > 0.05).

## Data Availability

The data presented in this study are available in the article.
